# Quantitative assessment of H&E staining for pathology: development and clinical evaluation of a novel system

**DOI:** 10.1186/s13000-024-01461-w

**Published:** 2024-02-23

**Authors:** Catriona Dunn, David Brettle, Martin Cockroft, Elizabeth Keating, Craig Revie, Darren Treanor

**Affiliations:** 1 National Pathology Imaging Co-operative, Leeds Teaching Hospitals NHS Trust, Leeds, UK; 2 Department of Pathology and Data Analytics, University of Leeds, Leeds, UK; 3 New Technology Group, Futamura Chemical UK Limited, Wigton, UK; 4 FFEI Limited, The Cube, Hemel Hempstead, UK; 5 Department of Histopathology, Leeds Teaching Hospitals NHS Trust, Leeds, UK; 6 Department of Clinical Pathology and Clinical and Experimental Medicine, Linköping University, Linköping, Sweden; 7 Centre for Medical Image Science and Visualisation, Linköping University, Linköping, Sweden

**Keywords:** Digital Pathology, Histopathology, Quality, Stain, Quality Assurance, Histochemical staining

## Abstract

**Background:**

Staining tissue samples to visualise cellular detail and tissue structure is at the core of pathology diagnosis, but variations in staining can result in significantly different appearances of the tissue sample. While the human visual system is adept at compensating for stain variation, with the growth of digital imaging in pathology, the impact of this variation can be more profound. Despite the ubiquity of haematoxylin and eosin staining in clinical practice worldwide, objective quantification is not yet available. We propose a method for quantitative haematoxylin and eosin stain assessment to facilitate quality assurance of histopathology staining, enabling truly quantitative quality control and improved standardisation.

**Methods:**

The stain quantification method comprises conventional microscope slides with a stain-responsive biopolymer film affixed to one side, called *stain assessment slides*. The stain assessment slides were characterised with haematoxylin and eosin, and implemented in one clinical laboratory to quantify variation levels.

**Results:**

Stain assessment slide stain uptake increased linearly with duration of haematoxylin and eosin staining (*r* = 0.99), and demonstrated linearly comparable staining to samples of human liver tissue (r values 0.98–0.99). Laboratory implementation of this technique quantified intra- and inter-instrument variation of staining instruments at one point in time and across a five-day period.

**Conclusion:**

The proposed method has been shown to reliably quantify stain uptake, providing an effective laboratory quality control method for stain variation. This is especially important for whole slide imaging and the future development of artificial intelligence in digital pathology.

**Supplementary Information:**

The online version contains supplementary material available at 10.1186/s13000-024-01461-w.

## Introduction

The histopathological examination of tissue is the cornerstone of cancer diagnosis globally. It is based on the staining of tissue samples with histochemical dyes, such as haematoxylin and eosin (H&E), to highlight cellular components for visual interpretation by pathologists. This process has not changed for over a century, and it is well understood that there are variations in the method [1–5]. Staining variation is widely seen in clinical practice in pathology, both within and between laboratories [5–7]. Although not often highlighted as a clinical risk, detailed evidence in this area is lacking. Professional guidelines and laboratory practice emphasise the need to maintain stain quality and reduce variation through internal and external quality assessment, but routine quantitative assessment of H&E staining has to date been unachievable [8–10].

The need to quantify and control stain quality is given greater impetus with the increasing use of digital pathology. This is the process of scanning a glass pathology slide with a whole slide imaging system to produce a digital image. The technology has been promoted and adopted as it has the potential to improve workflow and quality in pathology services [11–13]. Its utilisation is growing due to the increasing maturity of whole slide imaging systems, displays, data handling and storage, significant clinical need for pathology service globally, and the use of artificial intelligence (AI) to augment human diagnosis [10, 14, 15].

Image quality, specifically colour, is an important parameter for AI as differences in colour are used to set thresholds to detect objects and patterns, meaning variation in the stained colour of tissue can impact upon AI algorithm performance. An increasing number of papers in the literature highlight the importance of colour stability for AI [5–7, 16–19]. To help mitigate the effect of stain variation, computer assisted methods can be employed such as s*tain normalisation*, the digital normalisation of an image’s colour, and *data augmentation*, where computer simulated images with variable staining are introduced to training datasets to improve AI robustness [20–23]. With *stain normalisation*, the accuracy of AI, before and after normalisation, has been shown to deliver significant improvements in AI performance [19, 23–27]. Examples include improving colorectal cancer classification and prostate cancer detection accuracy by 20% and 9% respectively [26, 27]. Other work has found that prostate cancer classification performance suffered when using images from different institutions and scanners, and that application of stain normalisation to a variable-quality dataset improved AI performance by 5% [24]. Inter-institutional staining characteristics can be distinguishable by AI and have the potential to bias accuracy, even with application of stain normalisation [7]. Importantly, a recent study also found stain normalisation significantly improved pathologist perception of stain colour quality, diagnostic confidence, and time to diagnosis [28]. However, they also found that normalisation reduced inter-pathologist agreement. Although this was only two pathologists it suggests that normalisation may improve perceived colour and pathologist confidence, but that the normalisation process may be a variable in its own right that could negatively impact upon inter-observer agreement. Stain normalisation can improve image standardisation, AI performance and generalisability, however image manipulation is relative and can introduce artefacts, lead to loss of information, or bias the training data [23, 29–31].

An alternative approach to reduce variation between images is to reduce stain variation at its source, through laboratory quality control (QC). Strict protocols are maintained within histopathology laboratories and reagents are replenished regularly to minimise variation, with most adopting automated staining instruments for improved precision. Methods of routine QC have changed little over the years, where both internal and external quality assessments are based on subjective, *qualitative* observations [5, 32–35]. Although human qualitative assessment is important for assessing quality, it is subject to observer bias and relies on assessing stained control tissue which, due to intrinsic biological differences, can be variable between sections. Control tissue blocks are finite, being exhausted after a few hundred control sections have been cut, necessitating new controls which may have different morphological appearances and staining characteristics. Tissue staining may also be confounded by other variables prior to staining, such as fixation or section thickness variation [36–39]. These limitations mean that using tissue-based QC approaches alone may not be sufficient as a control method for stain quality assessment over time, or across institutions.

There has been research into the use of quantitative controls for *immunohistochemistry* staining in histopathology, and a consortium has recently been launched to improve immunohistochemistry reproducibility [40–44]. But there is limited research focusing on quantitative QC methods for *H&E* staining, which accounts for the majority of stained slides in laboratories worldwide. Gray et al. [5] and Chlipala et al. [45] have developed digital methods of quantifying H&E staining from whole slide images of stained control tissue. Although effective, these methods of quantifying stain can be impacted by confounding variables as they use tissue as a control and rely on accurate colour reproduction during digitisation.

In this paper we propose a method for absolute quantification of H&E staining in the laboratory environment, using *stain assessment slides.* Stain assessment slides comprise of a biopolymer film applied as a label to standard pathology glass slides. The biopolymer film is highly receptive to stain due to its hydrophilicity and porous structure. We characterise the stain assessment slides, compare the stain response with tissue, and validate the use of this methodology as routine QC testing for H&E staining within a clinical laboratory. This technique has the potential to offer truly objective and quantitative QC of H&E staining, to augment current QC processes in laboratories.

## Materials and methods

### Experiment 1: stain assessment slide H&E characterisation

#### Methodology

**Fig. 1:**
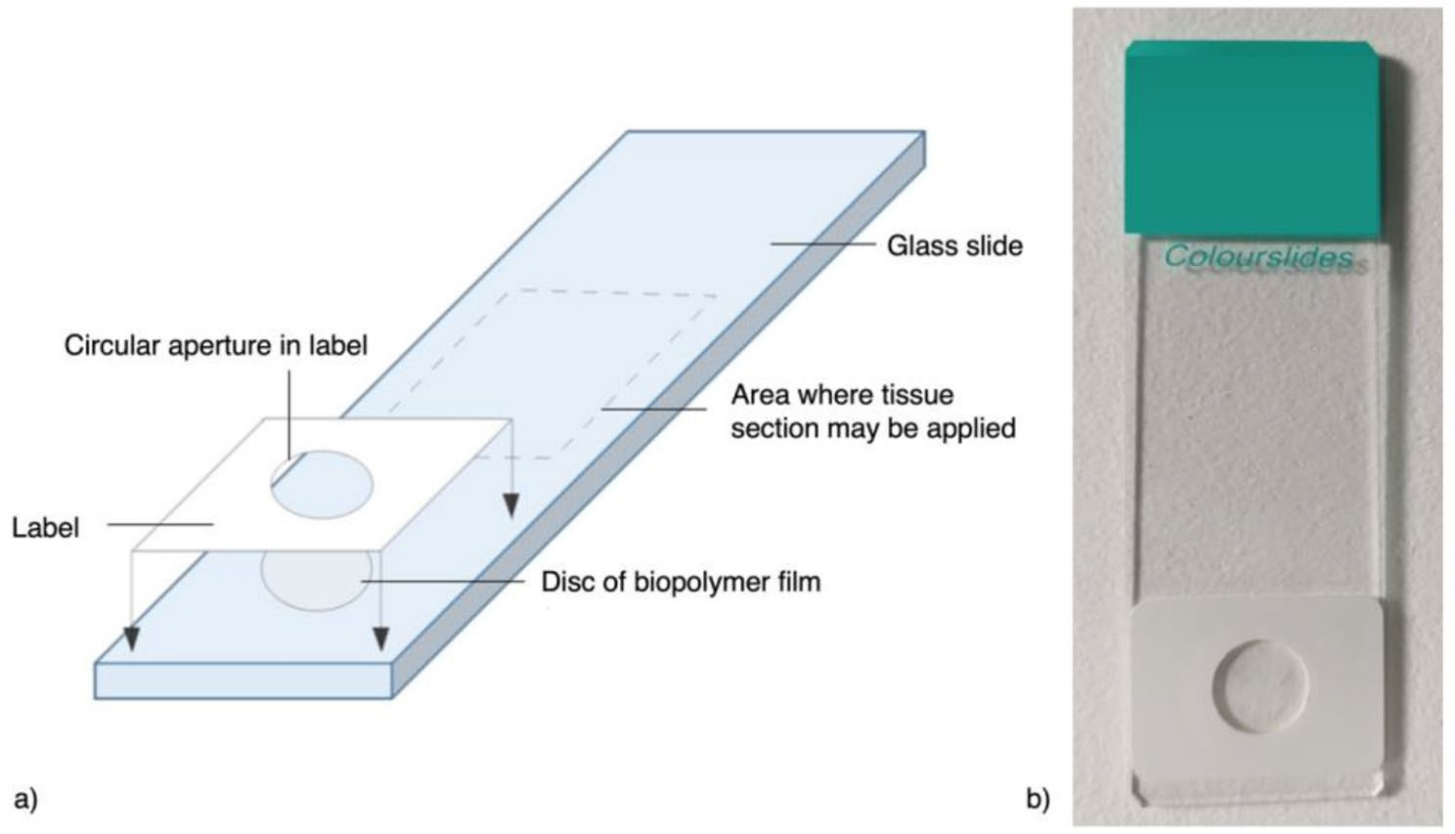
Stain assessment slide. An illustration **(a)** and a photo **(b)** of an example stain assessment slide, consisting of a disc of biopolymer film positioned onto a glass slide, with a chemically resistant PET label positioned on top to affix the biopolymer in position. The dotted grey line indicates the area where tissue sections may be mounted

A biopolymer film, with a standard thickness of 24.4 μm (±2%), was sourced (Futamura Chemical UK Limited, Wigton, UK). Discs of the biopolymer film (10 mm diameter) were cut and positioned onto non-coated glass slides (25 x 50 mm; Solmedia Ltd, Shrewsbury, UK). Chemically resistant polyethylene terephthalate (PET) labels with acrylic adhesive (17 x 25 mm; North and South labels Ltd, Thornton Heath, UK) had a central 8 mm diameter circular aperture removed and were overlaid to adhere the biopolymer film to the slides. Hereafter these slides will be referred to as *stain assessment slides*; they are depicted in Fig. 1.

Stain assessment slides were manually stained with Mayer’s haematoxylin and eosin Y 1% aqueous (see Supplementary Information Table 1 for information on stains and reagents used) according to the protocol in Table 1. Three stain techniques were used: (1) haematoxylin-only, (2) eosin-only and (3) H&E combined (equal stain duration for each stain). For each stain technique slides were stained for 13 stain durations, from 15 s to 6 min, with five slides at each stain duration (*n* = 65 per stain technique). Stain durations are shown in Supplementary Information Table 2.

**Table 1:** Staining protocol used in Experiments 1 and 2

Process	Step	Solution	Duration (m:s)		Process	Step	Solution	Duration (m:s)
**Dewaxing**	1	Xylene (wash 1)	3:00		**Staining**	14	Running tap water	1:00
	2	Xylene (wash 2)	3:00			15	Scott’s Tap Water	2:00
	3	Xylene (wash 3)	3:00			16	Running tap water	1:00
	4	Xylene (wash 4)	3:00			17	Aqueous Eosin Y (1%)	0:15 − 6:00 (see SI Table 2)
**Rehydration**	5	100% ethanol (wash 1)	3:00			18	Running tap water	1:00
	6	100% ethanol (wash 2)	3:00		**Dehydration**	19	100% ethanol (wash 1)	0:30
	7	100% ethanol (wash 3)	3:00			20	100% ethanol (wash 2)	1:00
	8	100% ethanol (wash 4)	3:00			21	100% ethanol (wash 3)	5:00
	9	75% ethanol	3:00			22	100% ethanol (wash 4)	5:00
	10	50% ethanol	3:00		**Clearing**	23	Xylene (wash 1)	3:00
	11	25% ethanol	1:00			24	Xylene (wash 2)	3:00
	12	Running tap water	2:00			25	Xylene (wash 3)	3:00
**Staining**	13	Mayer’s Haematoxylin	0:15 − 6:00 (see SI Table 2)		**Mounting**	26	DPX mountant	-

The staining protocol used in Experiment 1 and 2 is described, outlining process, solution and duration used. All steps in this protocol were undertaken at room temperature. Please note stain technique 1 (haematoxylin only) excluded steps 17–18, and stain technique 2 (eosin only) excluded steps 13–16. Stain technique 3 included all steps. Abbreviations: m:s, minutes : seconds; SI, Supplementary Information; DPX, Dibutylphthalate Polystyrene Xylene

#### Analysis

The stain assessment slides were scanned in a UV-Vis Cary100 spectrophotometer (Agilent Technologies, Santa Clara, USA). Prior to scanning, the spectrophotometer was calibrated using certified reference materials traceable to the National Physical Laboratory (Teddington, UK) primary references, and the baseline and zero were set following the standard procedure [46–48]. Absorbance spectra were measured from each slide between 350 and 800 nanometres (nm), at 1 nm increments. Total absorbance was calculated from each spectrum to provide a single number for comparison between slides. Total absorbance was the sum of all absorbance values within the visible spectrum (380–740 nm). Average total absorbance was calculated for each stain duration and technique, and plotted onto a scatter graphs with linear trend-lines applied. Error bars of one standard deviation from the mean were included. Using Minitab Desktop 21.2 statistical software (State College, USA), Pearson’s correlation coefficient (*r*) was calculated to assess the strength of the linear relationship, and coefficient of variation (C_V_) was calculated to show relative standard deviation(σ) as a percentage of the mean (µ), using Eq. (1). The C_V_ was calculated for each stain duration and averaged for each stain technique with 95% confidence intervals provided for each C_V_ average.

1 $$Cv= \frac{\sigma }{\mu }\times 100$$

### Experiment 2: characterisation with tissue

#### Methodology

55 stain assessment slides were constructed using the technique described in Experiment 1. To allow for increased space on the slide for tissue mounting, this experiment used 4 mm discs of biopolymer, overlaid with the PET label cut to smaller dimensions, 4.5 × 7.5 mm, with a 2 mm circular aperture removed.

Surplus human liver tissue was sourced. The tissue was processed using a Leica ASP300S processor (Leica Biosystems, Wetzlar, Germany) and embedded into a paraffin wax block. The tissue was sectioned to 5 μm using a microtome by a senior research technician and mounted onto the stain assessment slides above the biopolymer label (see Fig. 1). The slides were placed onto a hot plate at 60 °C for two hours and stained using Mayer’s haematoxylin and eosin Y aqueous 1% (equal time each stain, see Supplementary Information Table 1 for stain information) according to the protocol in Table 1, for stain durations between 15 s and 6 min (see Supplementary Information Table 2), with five slides stained at each stain duration (*n* = 55).

#### Analysis

The slides were scanned in an Aperio AT 2 whole slide imaging scanner (Leica Biosystems) at 20x magnification (0.5 microns per pixel), with JPEG compression (quality = 70). Digital images were used in this experiment, as opposed to spectral measurements, to enable an average colour measurement across the entire biopolymer and tissue area (excluding areas with artefacts, such as folds), to determine the relative relationship. The scanned images were viewed on Aperio ImageScope 12.1 (Leica Biosystems) and extracted, using the extract region tool, as jpeg files using JPEG2000 compression (quality score 30). The extracted images were viewed on ImageJ (Bethesda, Maryland, USA), where colour was measured in Red (R), Green (G) and Blue (B) – RGB – colour space. In this colour space, R is a numerical representation of the stained colour intensity in the red spectrum, G in the green, and B in the blue. Median RGB values of biopolymer and tissue on each slide were calculated and plotted against each other on a scatter graph. Using Minitab, Pearson’s correlation coefficient (*r*) was calculated to assess the strength of any correlation and C_V_ was calculated for each stain duration and averaged, with 95% confidence intervals provided.

### Experiment 3: clinical implementation

To validate the stain assessment slides as a QC method, two proof of concept studies were conducted in one clinical laboratory using automated staining instruments. The two arms to this experiment were (a) assessment of variation at one point in time, and (b) assessment of variation over a five-day period. Three clinically active staining instruments of the same manufacturer and model were tested; assigned as Stainer-1, Stainer-2 and Stainer-3. All instruments used identical H&E staining protocols.

### Methodology

#### Experiment 3a) assessment of variation at one point-in-time

This assessment was undertaken to test the level of variation of the stain assessment slides within three instruments at one point-in-time. 90 stain assessment slides were constructed, as described in Experiment 1. One full rack of slides (*n* = 30) was positioned in each of three staining instruments (Stainer-1, Stainer-2 and Stainer-3) and stained at one point-in-time, using the laboratory’s standard H&E staining protocol.

#### Experiment 3b) assessment of stain variation over five days

For assessment of variation within staining instruments over time, the three staining instruments were assessed over a period of five days. 75 stain assessment slides were constructed using the method described in Experiment 1. One stain assessment slide was placed in each of the three staining instruments and stained with H&E alongside tissue samples for routine clinical diagnosis. This was repeated five times per day, over a period of five days (Monday to Friday). The time of staining was spread across each day between 9:00 and 17:00 h.

### Analysis

#### Experiments 3a and 3b

After staining, the stain assessment slides from Experiment 3a and 3b were scanned in a spectrophotometer as detailed in Experiment 1. From the absorbance spectra, total absorbance was calculated. Using Minitab, boxplots were generated showing the spread of results. For Experiment 3a, C_V_ was calculated to measure *intra*-instrument and *inter*-instrument variation at one point in time. For Experiment 3b C_V_ was calculated to measure *intra*-instrument variation for individual days and across the five days, and *inter*-instrument variation across the five days, with 95% confidence intervals provided.

Inter-instrument variation in Experiment 3a and 3b was found to be normally distributed using the Anderson-Darling normality test and so analysis of variance (ANOVA) tests were carried out on the data, where *p* < 0.05 is considered significant, to compare results for inter-instrument variation across five days and at one point in time.

## Results

### Experiment 1: stain assessment slide characterisation

**Fig. 2:**
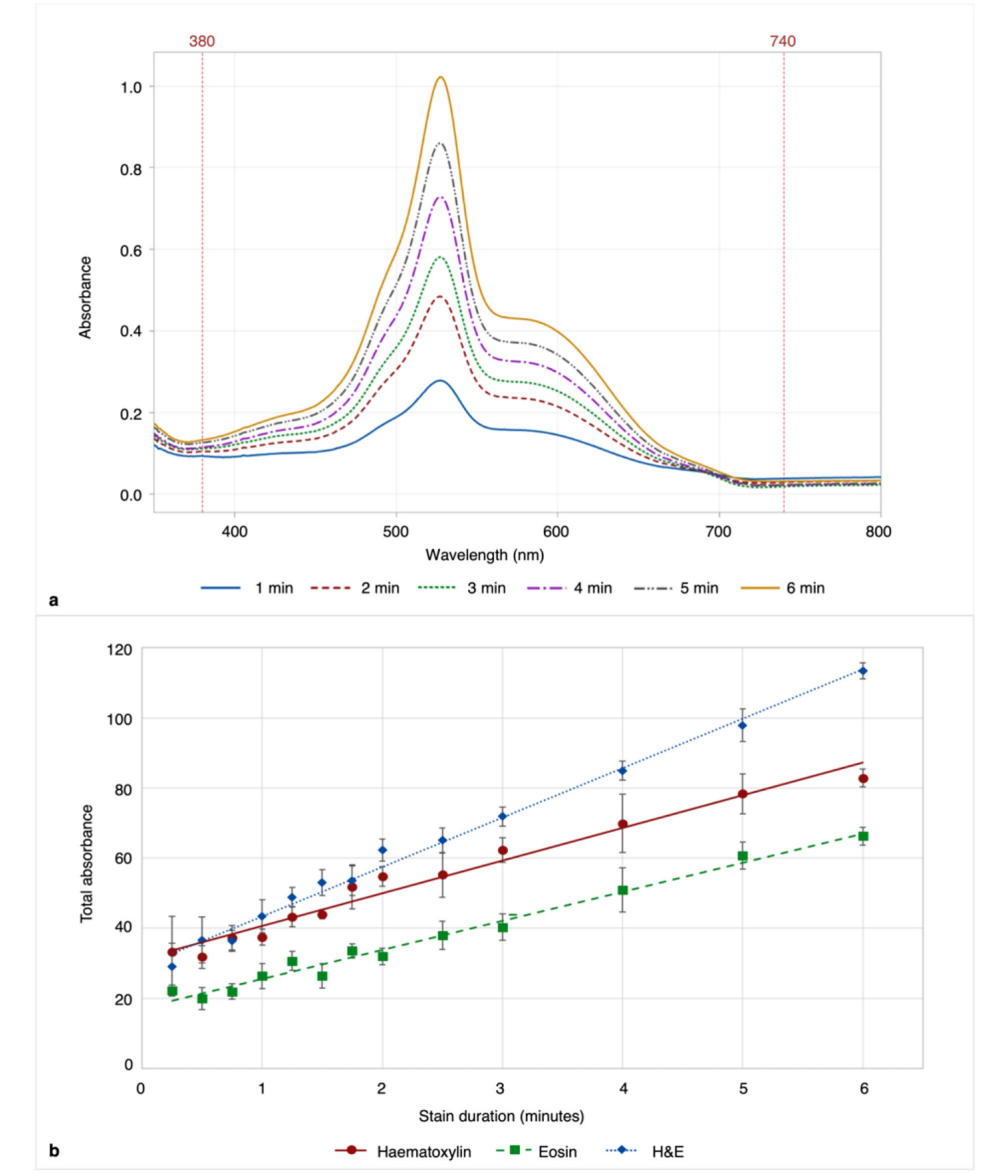
Stain assessment slide H&E stain response. **a)** Mean absorbance spectra of biopolymer film on stain assessment slides, stained with H&E (equal time each stain) from 1 to 6 min, with five slides stained at each stain duration. The reference lines provided indicate the portion of the spectrum that represents visible light wavelengths, between 380 and 740 nm, from which total absorbance was measured. **b** Average total absorbance of biopolymer film stained using haematoxylin, eosin and H&E combined, for durations ranging from 15 s to 6 min. Each point plotted is the average of five slides at each stain duration, with error bars depicting one standard deviation from the mean in each direction and linear trend lines applied

Figure 2a shows an example of six averaged spectra from H&E-stained stain assessment slides, with sparse data shown for clarity (stain durations: 1–6 min). The spectra demonstrate that as stain duration increased, the portion of the spectral curve, where the biopolymer absorbed light, increased incrementally for each stain technique, indicating increasing intensity.

Average total absorbance within the visible spectrum (380–740 nm, as highlighted between the reference lines in Fig. 2a for each stain duration and technique were plotted in Fig. 2b. Average total absorbance for each stain technique increased linearly over time, with Pearson’s correlation coefficient (*r)* values of 0.99 (haematoxylin-only), 0.99 (eosin-only) and 0.99 (H&E). Error bars depicting one standard deviation from the mean at each time point highlight the variation between samples. The average C_V_, with 95% confidence intervals displayed as (lower limit, upper limit), for all time durations was 11% (6, 16), 11% (9, 13) and 9% (5, 13) for haematoxylin-only, eosin-only and H&E combined respectively. See Supplementary Information Table 3 for the full range of standard deviation and C_V_ values for each stain duration and technique.

### Experiment 2: comparison of stain assessment slides and tissue

**Fig. 3:**
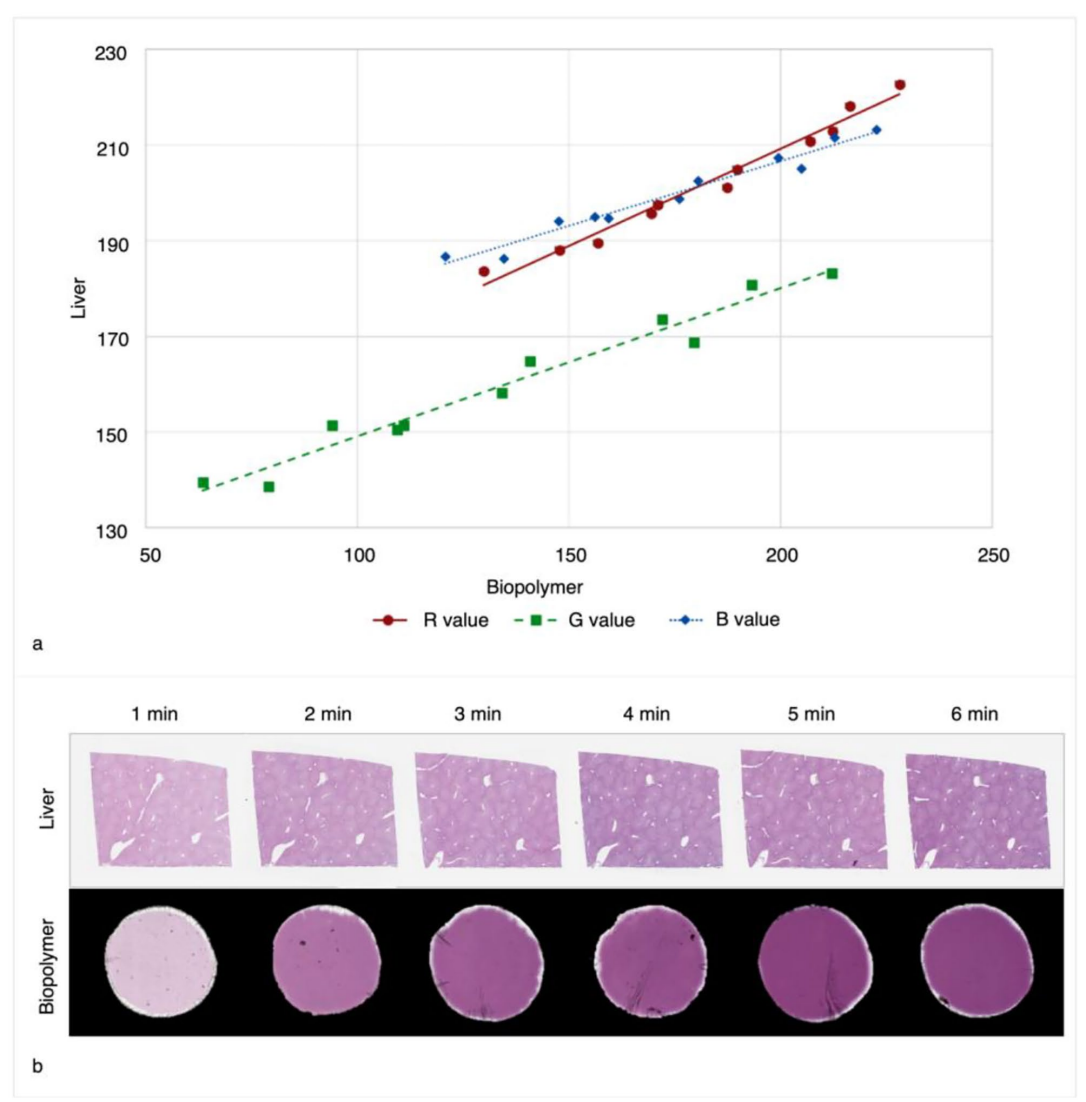
H&E stain response of stain assessment slides and human liver tissue. **(a)** Stain response scatterplot comparing median Red (R), Green (G) and Blue (B) colour values of human liver tissue against biopolymer film on stain assessment slides, stained with H&E between 15 s and 6 min (equal duration for each stain). Five slides were stained at each stain duration, with linear trend-lines applied. **(b)** Thumbnail images for visual comparison of stain response measured from whole slide images of human liver tissue and biopolymer film, stained with H&E from 1 – 6 min (equal duration for each stain)

Median RGB values of H&E stained biopolymer and human liver tissue are plotted against each other in Fig. 3a. Figure 3b provides thumbnail images of liver and biopolymer stained with H&E for 1–6 min, for visual comparison of stained colour. There was a linear correlation found between the biopolymer and liver tissue for R (*r* =0.99), G (*r* =0.98) and B (*r* =0.98) values. The average C_V_ of all the stain durations for RGB values respectively was 2% (1, 2), 4% (3, 5) and 2% (1, 2) for liver tissue, and 6% (5, 8), 14% (10, 18) and 7% (5, 10) for the biopolymer.

### Experiment 3: clinical implementation

#### Experiment 3a) assessment of variation at one point in time

**Fig. 4:**
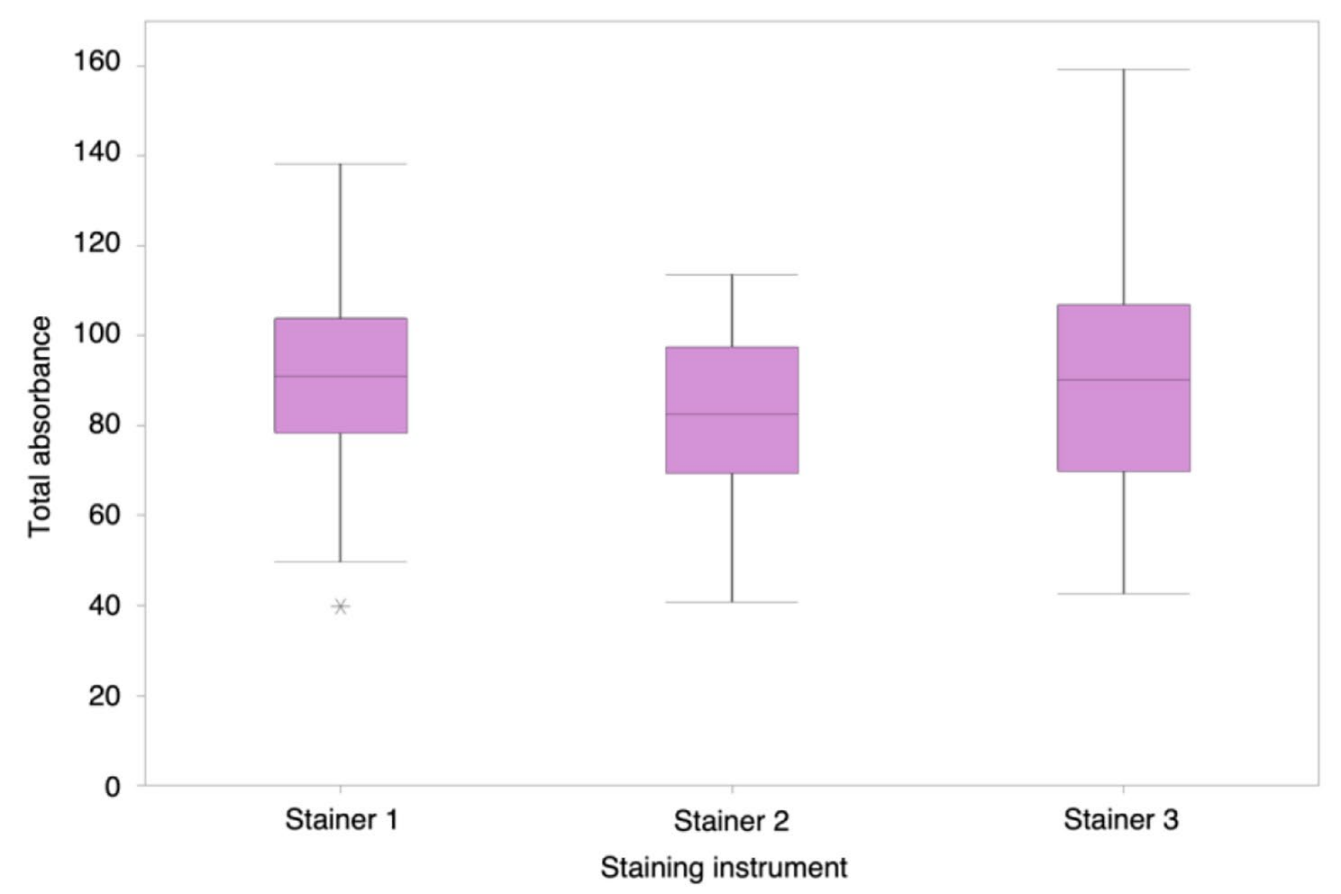
Spread of total absorbance across a five day period. Boxplots showing the spread of results of total absorbance measured from absorbance spectra of stain assessment slides stained in three staining instruments in a clinical laboratory during Experiment 3b. Five slides were stained in each stain instrument per day, using the same staining protocol, over a period of five days (*n* = 25 per stain instrument). Stainer-1 box is 79 – 104, whiskers are 50 – 138 with median of 91 and an outlier at 40. Stainer-2 box is 70 – 97, whiskers are 41 – 114 with a median of 83. Stainer-3 box is 70 – 107, whiskers are 43 – 159 with a median of 90

Boxplots showing the spread of total absorbance measured from the stain assessment slides for each instrument at one point in time can be seen in Fig. 4a. Intra-instrument variation (C_V_) was 6% (Stainer-1), 9% (Stainer-2) and 7% (Stainer-3), showing the level of variation in stain assessment slides at one point in time. The inter-instrument variation (C_V_) at one point in time was 8%. This variation was calculated to be statistically significant (*p* = 0.0003).

#### Experiment 3b) Assessment of daily stain variation

Boxplots showing the spread of total absorbance for each instrument across five days can be seen in Fig. 4b. The C_V_ across the five days was 28% (Stainer-1), 23% (Stainer-2) and 30% (Stainer-3), indicating the intra-instrument variation for each stain instrument over the time-period. The intra-instrument variation over five days was statistically significant for Stainer-2 (*p*=0.001), but not significant for Stainer-1 (*p*=0.699) or Stainer-3 (*p*=0.062). The inter-instrument variation (C_V_) was 27%, but this was not statistically significant (*p*=0.441). See Supplementary Information Table 4 for more detailed results from Experiment 3a and 3b.

## Discussion

We have proposed that improving stain QC and standardisation is a practical and logical approach to ensuring consistency of traditional laboratory stain quality and the resultant digital data set.

We evaluated a novel method of stain QC in a series of experiments. Experiment 1 characterised the biopolymer film on stain assessment slides, stained with H&E (separately and combined) and found a linear relationship between stain duration and stained colour of the biopolymer, with *r* values of 0.99 for all stain techniques. This demonstrated that the stain assessment slides take up H&E stain linearly over time and were an effective, quantitative measure of staining, based on purposefully altering stain duration.

Experiment 2 compared the H&E staining characteristics of the biopolymer with sections of human liver tissue, to contrast the performance of the system with the conventional use of tissue-based controls. There was a strong correlation between mean biopolymer and liver staining (*r* values between 0.98 and 0.99) indicating that biopolymer stain uptake was linearly comparable to human liver tissue within the stain durations measured. The linear relationship was non-proportional (y intercept ≠ 0) due to the biopolymer film having an increased thickness (24.4 μm biopolymer vs. 5 μm tissue sections), permitting higher sensitivity of the biopolymer to detect variations in staining.

Experiment 3 implemented stain assessment slides within a clinical laboratory to establish the clinical utility of the method. Experiment *3a* assessed variation at one point in time and found the intra-instrument variation was 6–9%; a similar level to the average variation found across stain durations in Experiments 1 and 2. This suggests that this variation was dominated by intra-batch variation in the stain assessment slides, rather than variation within the staining instruments, however this was not possible to discriminate. The inter-instrument variation at one point in time was 8%, which was found to be statistically significant (*p* = 0.0003). This indicated that despite different instruments using the same protocol, inter-instrument variations are present. Varying levels of slide throughput may have contributed to this, e.g. a higher throughput of slides may equate to a higher likelihood of reagents becoming diluted/contaminated. There may also be variations between different H&E stain batches that could contribute to the variation measured. The stain assessment slides offer a simple method of quantifying variation and characterising staining instruments on a periodic basis. However, despite the instruments staining being significantly different, only 8% variation was measured at one point in time, which was a low level of variation (similar to baseline level of variation within the stain assessment slides), particularly considering the biopolymer has an increased sensitivity to stain compared to human tissue.

Experiment *3b* assessed variation across five days and found an average intra-instrument variation of between 23 and 28%. This is approximately 2.5–4.5 times higher than the level of variation found in Experiment *3a* at one point in time, which highlights the increased variation present across five days. The daily variation reached as high as 47% on one day (Stainer-3, day 2). The inter-instrument variation was 27% but was not found to be significant, although this may be due to paucity of data. The variation was likely caused by dilution of reagents and high throughput of slides over the course of one week. Daily quantitative QC would have a strong potential to limit this variation by setting thresholds of normal operation; this would also provide onward benefits for AI by providing more consistent data for both training and utilisation. A limitation of this experiment was that information was not collected on the frequency of stain reagent changes. As one of the potential benefits of the stain assessment slides would be to optimise reagent use, that information is important and should be included in future work. If less frequent reagent changes can be identified this could be of financial benefit to laboratories, either way this information potentially informs on future guidelines or standards.

Additional limitations of this study include the variability in stain uptake by the biopolymer at 6–14%. For context this variability was subjectively barely perceivable compared to the staining instrument variation found across five days, which was readily noticeable at 23–28%. It is thought that the variation was largely due to the high sensitivity of the biopolymer; the hand-made nature of constructing stain assessment slides; and the use of a manual staining process in Experiments 1 and 2. As such automated manufacture and staining processes should improve this. A further limitation was the use of different techniques to measure the colour of the biopolymer film. In Experiments 1 and 3, colour was measured spectrally (total absorbance), to characterise the *absolute* stained colour in the biopolymer. Experiment 2 differed in that colour was measured digitally (RGB values) to characterise the *relative* relationship between the biopolymer and tissue stain uptake. Accurate colour measurement from whole slide images relies on accurate colour reproduction of the imaging system. The whole slide images were manually checked for quality, but the AT 2 scanner was not specifically colour-calibrated prior to use, other than the out-of-factory calibration, setup and yearly calibration by the manufacturers following their standard procedure. Because we scanned the biopolymer and tissue in the same scanner at the same time, we can determine from previous experimental work that this scanner would have an expected variation in colour measurement of 0.47%, which is an order of magnitude lower than the stain variation being measured in the stain assessment slides and tissue [5]. There was no direct comparison between the spectral and digital colour measurements and future work will compare these methods.

The H&E characterisation in this paper was based on an intensity measurement of H&E staining with equal time for each stain (1:1 ratio), so additional analysis is needed to understand the biopolymer response to disproportionate H&E stain durations. Early work suggests that this will be proportional to the time-stain uptake curves shown in **Fig. 2b**. The relative uptake of H&E stains needs to be reported to inform practical instrument optimisation in the laboratory. Digital methods do exist to do this already, for example, stain deconvolution by Ruifrok et al. [49]. The impact of varying H&E types/brands also needs to be fully characterised, as well as determination of the level of variation in stain assessment slides that equates to visually or diagnostically noticeable differences in different tissue types.

Further work will develop an operational process to allow stain assessment slides to be readily deployed and utilised in an operational environment. The use of a spectrophotometer is impractical in an operational pathology workflow, however if a laboratory has been digitalised already, a whole slide imager could practically be used to collect stain data. There are two potential limitations of this, one is that not all laboratories have gone digital, and the other is that a time lag is introduced between staining and the returned quantitative data, which may limit the utility of the stain assessment tool as a near-time quality control. To address this, we are developing a small, laboratory-friendly device to measure colour directly from the stain assessment slides that can fit easily into the laboratory workflow and provide immediate feedback. It is important to note that the stain assessment slides allow quantification of the stain delivered to tissue. We accept that there are complex relationships between haematoxylin, eosin and tissue presentation. The use of stain assessment slides is not for assessing the impact on clinical presentation, but to provide information that the staining instrument may or may not be performing within pre-defined parameters as that may have a consequence for the clinical presentation.

In summary, this work presents a novel method using a biopolymer as a quantitative H&E stain assessment tool that:

- demonstrates linear staining with H&E,
- shows comparable stain uptake to control tissue slides,
- has demonstrable clinical utility in measuring stain variation.

If adopted into routine practice, the presented QC tool could improve stain consistency and optimise reagent use by removing subjectivity in stain assessment. This technique can be used as a periodic point-in-time test for staining instruments, to be used alongside laboratory internal and external qualitative assessment protocols. An added benefit of quantifying stain variability is the potential cost-saving by optimising stain replenishment and reducing reagent use. There are also clinical and operational benefits from reducing the need to re-section and re-stain tissue if stain quality drops. These benefits will not only help optimise the speed and quality of diagnosis but also help to produce consistent digital whole slide images and to help facilitate AI in digital pathology in future.

### Electronic supplementary material

Below is the link to the electronic supplementary material.

Supplementary Material 1

Supplementary Material 2

## Data Availability

The datasets supporting the conclusions of this article are included within the articles Supplementary Information.

## Abbreviations

ANOVA: Analysis of variance
AI: artificial intelligence
C_V_: coefficient of variation
DPX: Dibutylphthalate Polystyrene Xylene
H&E: haematoxylin and eosin
µ: mean
m: s:minutes:seconds
nm: nanometres
PET: polyethylene terephthalate
QC: quality control
RGB: red, green and blue
σ: standard deviation
SI: supplementary information

## References

[1] Titford M. A Short History of Histopathology Technique, *Journal of Histotechnology*, vol. 29, no. 2, pp. 99–110,2006/06/01 2006, 10.1179/his.2006.29.2.99.

[2] Hussein I, Raad M, Safa R, Jurjus RA, Jurjus A. Once upon a microscopic slide: the story of histology. J Cytol Histol, 6, 2015.

[3] Lyon HO et al. Standardization of reagents and methods used in cytological and histological practice with emphasis on dyes, stains and chromogenic reagents. Histochemical J J Article 26, 7, pp. 533–44, July 01 1994, 10.1007/bf00158587. 10.1007/BF00158587 7525512

[4] Bejnordi BE, Timofeeva N, Otte-Höller I, Karssemeijer N. W. M. v. d. Laak, quantitative analysis of stain variability in histology slides and an algorithm for standardization. Med Imaging 2014: Digit Pathol. 2014;9041. 10.1117/12.2043683.

[5] Gray A, Wright A, Jackson P, Hale M, Treanor D. Quantification of histochemical stains using whole slide imaging: development of a method and demonstration of its usefulness in laboratory quality control, (in eng). J Clin Pathol. Mar 2014;68(3):192–9. 10.1136/jclinpath-2014-202526. 10.1136/jclinpath-2014-202526 25480984

[6] Bejnordi BE et al. Stain Specific Standardization of Whole-Slide Histopathological Images, (in eng), *IEEE transactions on medical imaging*, vol. 35, no. 2, pp. 404 – 15, Feb 2016, 10.1109/tmi.2015.2476509. 10.1109/TMI.2015.2476509 26353368

[7] Howard FM (2021). The impact of site-specific digital histology signatures on deep learning model accuracy and bias. Nat Commun.

[8] The Royal College of Pathologists., How to Assess the Quality of a Pathology Service, www.rcpath.org/uploads/assets/1c3aac02-3f31-4246-83b9da4aa04899ca/How-to-Assess-the-Quality-of-a-Pathology-Service-https://www.rcpath.org/uploads/assets/1c3aac02-3f31-4246-83b9da4aa04899ca/How-to-Assess-the-Quality-of-a-Pathology-Service-meeting-report.pdf 2011. [Online]. Available:

[9] Robert Lott JT, Sheppard E, Santiago J, Hladik C, Nasim M, Zeitner K, Haas T. Shane Kohl, Saeid Movahedi-Lankaran, Practical Guide to Specimen Handling in Surgical Pathology, <https://cap.objects.frb.io/documents/practical-guide-specimen-handling.pdf> College of American Pathologists, 2020. [Online]. Available: https://cap.objects.frb.io/documents/practical-guide-specimen-handling.pdf.

[10] Williams BJ, Knowles C, Treanor D (2019). Maintaining quality diagnosis with digital pathology: a practical guide to ISO 15189 accreditation. J Clin Pathol.

[11] Griffin J, Treanor D. Digital pathology in clinical use: where are we now and what is holding us back? *Histopathology*, vol. 70, no. 1, pp. 134–145, 2017. 10.1111/his.12993 27960232

[12] Gupta R, Kurc T, Sharma A, Almeida JS, Saltz J (2019). The emergence of pathomics. Curr Pathobiol Rep.

[13] Baxi V, Edwards R, Montalto M, Saha S. Digital pathology and artificial intelligence in translational medicine and clinical practice. Mod Pathol, 35, 1, pp. 23–32, 2022/01/01 2022, 10.1038/s41379-021-00919-2. 10.1038/s41379-021-00919-2 PMC8491759 34611303

[14] Niazi MKK, Parwani AV, Gurcan MN. Digital pathology and artificial intelligence. Lancet Oncol, 20, 5, pp. e253-e261, 2019/05/01/ 2019. 10.1016/S1470-2045(19)30154-8 PMC8711251 31044723

[15] Williams BJ, Bottoms D, Treanor D (2017). Future-proofing pathology: the case for clinical adoption of digital pathology. J Clin Pathol.

[16] Tellez D (2018). Whole-slide mitosis detection in H&E breast histology using PHH3 as a reference to Train Distilled Stain-Invariant Convolutional Networks. IEEE Trans Med Imaging.

[17] Schömig-Markiefka B et al. Quality control stress test for deep learning-based diagnostic model in digital pathology. Mod Pathol, 34, 12, pp. 2098–2108, 2021/12/01 2021, 10.1038/s41379-021-00859-x. 10.1038/s41379-021-00859-x PMC8592835 34168282

[18] Wright AI, Dunn CM, Hale M, Hutchins GGA, Treanor DE. The Effect of Quality Control on Accuracy of Digital Pathology Image Analysis, (in eng). IEEE J Biomed Health Inf. Feb 2021;25(2):307–14. 10.1109/JBHI.2020.3046094. 10.1109/JBHI.2020.3046094 33347418

[19] Bejnordi BE et al. Stain specific standardization of whole-slide histopathological images, (in eng). IEEE Trans Med Imaging, 35, 2, pp. 404 – 15, Feb 2015. 10.1109/TMI.2015.2476509 26353368

[20] Ciompi F et al. The importance of stain normalization in colorectal tissue classification with convolutional networks, 2017 *IEEE 14th International Symposium on Biomedical Imaging (ISBI 2017)*, pp. 160–163, 18–21 April 2017 2017, 10.1109/ISBI.2017.7950492.

[21] Khan AM, Rajpoot N, Treanor D, Magee D (2014). A nonlinear Mapping Approach to Stain normalization in Digital Histopathology images using image-specific Color Deconvolution. IEEE Trans Biomed Eng.

[22] Komura D, Ishikawa S (2018). Machine learning methods for histopathological image analysis. Comput Struct Biotechnol J.

[23] Tellez D et al. Quantifying the effects of data augmentation and stain color normalization in convolutional neural networks for computational pathology. Med Image Anal, 58, p. 101544, 2019/12/01/ 2019, doi: 10.1016/j.media.2019.101544. 10.1016/j.media.2019.101544 31466046

[24] Anghel A (2019). A high-performance system for robust stain normalization of whole-slide images in histopathology. Front Med.

[25] Swiderska-Chadaj Z (2020). Impact of rescanning and normalization on convolutional neural network performance in multi-center, whole-slide classification of prostate cancer. Sci Rep.

[26] Ciompi F et al. The importance of stain normalization in colorectal tissue classification with convolutional networks, in *2017 IEEE 14th International Symposium on Biomedical Imaging (ISBI 2017)*, 2017: IEEE, pp. 160–163.

[27] Salvi M, Molinari F, Acharya UR, Molinaro L, Meiburger KM (2021). Impact of stain normalization and patch selection on the performance of convolutional neural networks in histological breast and prostate cancer classification. Comput Methods Programs Biomed Update.

[28] Salvi M et al. Impact of Stain Normalization on Pathologist Assessment of Prostate Cancer: A Comparative Study, *Cancers*, vol. 15, no. 5, p. 1503, 2023. 10.3390/cancers15051503 PMC10000688 36900293

[29] Tosta TAA, de Faria PR, Neves LA, do Nascimento MZ (2019). Computational normalization of H&E-stained histological images: Progress, challenges and future potential. Artif Intell Med.

[30] Beena M (2016). A Survey on Color Normalization Approach to Histopathology images. Int J Adv Eng Res Sci.

[31] Howard FM et al. The Impact of Digital Histopathology Batch Effect on Deep Learning Model Accuracy and Bias, *bioRxiv*, 2020.

[32] Cox B, Colgan E. 1 - Pathology laboratory management, in *Bancroft’s Theory and Practice of Histological Techniques (Eighth Edition)*, S. K. Suvarna, C. Layton, and J. D. Bancroft Eds.: Elsevier, 2019, pp. 1–11.

[33] NSH. Histology Quality Improvement Program (HistoQIP). https://www.nsh.org/learn/histoqip (accessed 15/09/2020.

[34] UKNEQAS. UK National External Quality Assessment Service. https://ukneqas.org.uk (accessed.

[35] CAP. College of American Pathologists, Laboratory Accreditation Program. https://www.cap.org/laboratory-improvement/accreditation/laboratory-accreditation-program (accessed.

[36] Allison RT, Vincent JFV (1990). Measuring the forces acting during microtomy by the use of load cells. J Microsc.

[37] McCampbell AS (2019). Tissue thickness effects on immunohistochemical staining intensity of markers of Cancer. Appl Immunohistochem Mol Morphology.

[38] Bass BP, Engel KB, Greytak SR, Moore HM (2014). A review of preanalytical factors affecting molecular, protein, and morphological analysis of formalin-fixed, paraffin-embedded (FFPE) tissue: how well do you know your FFPE specimen?. Arch Pathol Lab Med.

[39] Chlipala EA (2021). Impact of preanalytical factors during histology processing on section suitability for digital image analysis. Toxicol Pathol.

[40] Hotzel KJ et al. Synthetic Antigen Gels as Practical Controls for Standardized and Quantitative Immunohistochemistry, (in eng), *The journal of histochemistry and cytochemistry: official journal of the Histochemistry Society*, vol. 67, no. 5, pp. 309–334, May 2019, 10.1369/0022155419832002. 10.1369/0022155419832002 PMC6495489 30879407

[41] Sompuram SR, Vani K, Tracey B, Kamstock DA, Bogen SA. Standardizing Immunohistochemistry: A New Reference Control for Detecting Staining Problems, (in eng), *The journal of histochemistry and cytochemistry: official journal of the Histochemistry Society*, vol. 63, no. 9, pp. 681 – 90, Sep 2015, 10.1369/0022155415588109. 10.1369/0022155415588109 PMC4804728 25940339

[42] Bogen SA et al. Experimental validation of peptide immunohistochemistry controls, (in eng), *Applied immunohistochemistry & molecular morphology: AIMM / official publication of the Society for Applied Immunohistochemistry*, vol. 17, no. 3, pp. 239 – 46, May 2009, 10.1097/PAI.0b013e3181904379. 10.1097/PAI.0b013e3181904379 PMC2672113 19077907

[43] Torlakovic EE (2021). Development and validation of measurement traceability for in situ immunoassays. Clin Chem.

[44] Bogen SA et al. A Consortium for Analytic standardization in immunohistochemistry. Arch Pathol Lab Med, 2022. 10.5858/arpa.2022-0031-RA PMC11681772 36084252

[45] Chlipala E (2020). Optical density-based image analysis method for the evaluation of hematoxylin and eosin staining precision. J Histotechnology.

[46] Allen DW (2007). Holmium Oxide Glass Wavelength standards, (in eng). J Res Natl Inst Stand Technol.

[47] Eckerle KL, Weidner VR, Hsia JJ, Kafadar K. Measurement Assurance Program Transmittance standards for Spectrophotometric Linearity Testing:* Preparation and Calibration. J Res Natl Bureau Stand, 88, 1, 1983. 10.6028/jres.088.003 PMC6768221 34566095

[48] National Physical Laboratory, A National Measurement Good Practise Guide. https://www.npl.co.uk/special-pages/guides/mgpg97 (accessed 09/10/19, 2019).

[49] Ruifrok AC, Johnston DA (2001). Quantification of histochemical staining by color deconvolution. Anal Quant Cytol Histol.

