# Supplementary material for: Quantitative assessment of H&E staining for pathology: development and clinical evaluation of a novel system
Source: Diagn Pathol. 2024 Feb 23;19:42. doi: 10.1186/s13000-024-01461-w (PMC10885446; doi:10.1186/s13000-024-01461-w)
Supplement: Supplementary file 1 — Supplementary Material 1 [file 13000_2024_1461_MOESM1_ESM.docx]

Supplementary information

**Supplementary Information Table 1** Solution and reagent details

| **Solution/reagent** |  |  |  | **Supplier** |
| --- | --- | --- | --- | --- |
| Xylene |  |  |  | Fisher Scientific UK Ltd (Loughborough, UK) |
| DPX |  |  |  | Solmedia Ltd (Shrewsbury, UK) |
| **Laboratory manual preparation of solution/reagent:** | | | | |
| **Solution/reagent** | **Step** | **Procedure** | **Components** | **Supplier** |
| Ethanol (different dilutions) | 1 | Dilute ethanol to different dilutions | 100% ethanol | Sigma-Aldrich (Missouri, USA) |
|  |  |  | Deionised water | N/a |
| Mayer’s haematoxylin | 1 | Solution 1 (mix together) | 6 g haematoxylin | Atom Scientific Ltd (Hyde, UK) |
|  |  |  | 40 mL 100% ethanol | Sigma-Aldrich |
|  | 2 | Solution 2 (add sequentially until dissolved) | 1700 mL distilled water | N/a |
|  |  |  | 0.6 g sodium iodate | VWR International |
|  |  |  | 2 g citric acid | Sigma-Aldrich |
|  |  |  | 100 g chloral hydrate | VWR International (Radnor, USA) |
|  |  |  | 100 g aluminium potassium sulphate | Sigma-Aldrich |
|  | 3 | Mix Solutions 1 and 2 together |  | |
|  | 4 | Add glycerol and mix (filter before use) | 240 mL glycerol | Sigma-Aldrich |
| Scott’s tap water | 1 | Mix together | 2 g sodium bicarbonate | Sigma-Aldrich |
|  |  |  | 20 g magnesium sulphate | Sigma-Aldrich |
|  |  |  | 1 L distilled water | N/a |
| 1% aqueous solution of eosin Y | 1 | Mix together (filter before use) | 10g eosin yellowish | Atom Scientific |
|  |  |  | 1L deionised water | N/a |

List of all solutions/reagents used, suppliers, and the composition and procedure used for those solutions/reagents that were manually prepared. Abbreviations: DPX, DPX, Dibutylphthalate Polystyrene Xylene; g, gram; mL, millilitre; L, litre

**Supplementary Information Table 2** Stain durations (m:s) used in Experiments 1 and 2

| **Slide number** | **1** | **2** | **3** | **4** | **5** | **6** | **7** | **8** | **9** | **10** | **11** | **12** | **13** |
| --- | --- | --- | --- | --- | --- | --- | --- | --- | --- | --- | --- | --- | --- |
| **Experiment 1** | 0:15 | 0:30 | 0:45 | 1:00 | 1:15 | 1:30 | 1:45 | 2:00 | 2:30 | 3:00 | 4:00 | 5:00 | 6:00 |
| **Experiment 2** | 0:15 | 0:30 | 0:45 | 1:00 | 1:30 | 2:00 | 2:30 | 3:00 | 4:00 | 5:00 | 6:00 | - | - |

Abbreviations: m:s, minutes : seconds

**Supplementary Information Table 3** Experiment 1 variation

| **Stain duration (m:s)** | **Technique 1** | | **Technique 2** | | **Technique 3** | |
| --- | --- | --- | --- | --- | --- | --- |
|  | **σ** | **C_v_** | **σ** | **C_v_** | **σ** | **C_v_** |
| **0:15** | 11.58 | 35% | 1.79 | 8% | 7.75 | 27% |
| **0:30** | 3.71 | 12% | 3.59 | 18% | 7.47 | 20% |
| **0:45** | 3.99 | 11% | 2.53 | 12% | 3.44 | 9% |
| **1:00** | 2.55 | 7% | 4.12 | 16% | 5.35 | 12% |
| **1:15** | 3.17 | 7% | 3.11 | 10% | 3.11 | 6% |
| **1:30** | 1.44 | 3% | 4.02 | 15% | 4.24 | 8% |
| **1:45** | 7.14 | 14% | 2.33 | 7% | 4.87 | 9% |
| **2:00** | 3.23 | 6% | 2.70 | 8% | 3.6 | 6% |
| **2:30** | 7.30 | 13% | 4.65 | 12% | 4.06 | 6% |
| **3:00** | 4.01 | 6% | 4.29 | 11% | 3.16 | 4% |
| **4:00** | 9.53 | 14% | 7.18 | 14% | 3.12 | 4% |
| **5:00** | 6.44 | 8% | 4.42 | 7% | 5.36 | 5% |
| **6:00** | 2.53 | 3% | 2.91 | 4% | 2.56 | 2% |

Standard deviation and coefficient of variation calculated from total absorbance of stain assessment slides from Experiment 1. Abbreviations: m:s, minutes : seconds

**Supplementary Information Table 4** Experiment 3 variation

|  | Day | Stainer-1 | | Stainer-2 | | Stainer-3 | | All stain instruments | |
| --- | --- | --- | --- | --- | --- | --- | --- | --- | --- |
|  |  | σ | C_v_ | σ | C_v_ | σ | C_v_ | σ | C_v_ |
| Experiment 3a | 1 | 7.42 | 6% | 9.89 | 9% | 7.75 | 7% | 9.12 | 8% |
| Experiment 3b | 1 | 38.82 | 45% | 9.92 | 16% | 25.07 | 33% | 27.24 | 36% |
|  | 2 | 13.78 | 18% | 16.93 | 24% | 31.5 | 47% | 21.07 | 29% |
|  | 3 | 1.91 | 2% | 7.39 | 7% | 4.7 | 4% | 5.14 | 5% |
|  | 4 | 39.13 | 42% | 19.95 | 23% | 12.36 | 13% | 24.66 | 27% |
|  | 5 | 12.21 | 13% | 8.07 | 9% | 30.79 | 29% | 18.96 | 19% |
|  | 1 - 5 | 25.01 | 28% | 19.02 | 23% | 26.49 | 30% | 23.67 | 27% |

Standard deviation and coefficient of variation calculated from total absorbance of stain assessment slides from Experiment 3a and Experiment 3b. Abbreviations: σ, standard deviation; C_v,_ coefficient of variation
